# High Dilutions and Their Effects from a Chemical Point of View

**Published:** 1898-06

**Authors:** J. Elliott Gilpin


### HIGH DILUTIONS AND THEIR EFFECTS, FROM A CHEMICAL POINT OF VIEW.

J. Elliott Gilpin, Ph. D.

Every physician has, no doubt, determined empirically the value and use of certain drugs, and knows that in his branch of science, as well as in all the natural sciences, explanations are not forthcoming for many observations and experiments which have been recorded. They know that small doses and those of high potencies are often efficient when stronger ones fail to act; but it remained for the chemist to discover related phenomena which may aid the physician to explain the cause of these results. These have been obtained through a study of the nature and properties of solutions. It will, perhaps, be well to review briefly the old idea of the nature of solutions and compare it with the present one. It was supposed that the action of the water, on salts dissolved in it, was of a physical nature consisting in the subdivision of the material into ultimate invisible particles, these being, perhaps, in a continual state of decomposition and recombination, without, however, remaining in the dissociated condition any conceivable length of time. According to this view the explanation of the fact that substances act upon each other more readily in solution than in the dry condition, was that the substances could come into more intimate contact with one another, and produce more rapid and complete action. There were many facts, however, some of which will be taken up later, which could not easily be explained by this theory. Some ten years ago Van't Hoff showed, by a study of the osmotic pressure of solutions, that all the laws governing the changes in volume and pressure of gases held equally well for dilute solutions of sugar and other substances. His conclusions were based on results obtained by Pfeffer in the study of the pressure exerted by substances in solution in water.

The apparatus used by this investigator consisted of a small clay cell, to the open end of which was attached a short piece of glass tubing. To this in turn was added a tube which would serve, on account of its small diameter, to indicate a small increase in pressure. The rise in the height of the column can be measured, or the pressure can be determined by connecting the small tube to a manometer. In order to allow the water free passage through the cell wall, but to prevent the passage of the dissolved substance, he precipitated a semi-permeable membrane within the pores of the cell, by saturating the cell with a solution of copper sulphate, then washing out the interior and introducing a solution of potassium ferrocyanide which would be absorbed by the porous cell and come in contact with the copper sulphate. The copper ferrocyanide precipitated forms a thin membrane, which while perfectly permeable to water, prevents the passage of the substance in solution. If now this cell is filled with a solution of cane sugar in water, and the whole cell introduced into a vessel containing distilled water, we will find in the course of some hours that the height of the liquid in the tube is greater, thus showing that the amount of liquid in the cell has increased. The membrane in the cell wall will allow the water to pass in or out with equal freedom; but for some reason which has not yet been clearly explained, the flow is greater into the cell, even though the molecules of the sugar are bombarding the membrane on the inner side, which one might expect would prevent the easy entrance of the water. If the quantity of material is determined and the increase in pressure observed, it will be found that the increase in pressure is that which the substance would exert if it were in the gaseous condition and occupied the same volume as the liquid. Other laws that hold for gases were found to hold equally well for dilute solutions, so that the analogy between gases and substances in solution seems well established. The exceptions to these laws were the acids, alkalies, and salts which are soluble in water; but these apparent exceptions were shown to be due to the fact that dissociation had taken place, giving rise to part-molecules or ions, which are greater in number than the undissociated molecules. Some idea of the increase in pressure that is due to a salt dissolved in water can be obtained from a consideration of the case of potassium nitrate, a 1 per cent. solution of which causes a pressure of three atmospheres. It was found also that the substances which did not have the power to transmit the electric current were the ones which gave normal values; while the electrolytes gave abnormal values so long as they were in solution in a solvent which had the power to dissociate them.

The conception that the passage of a current was intimately connected with the dissociation of the substance in solution was due to Faraday, who held the opinion that the current decomposed the substance, and that the ions thus set free transmitted the current through the liquid. The fact that a current that is too weak to cause any dissociation can still be transmitted through the liquid has led to a modification of the Faraday view, and according to the present electrolytic dissociation theory the ions exist in the free condition, even before the passage of the current, and the latter only influences the direction of the movements of the ions. According to this theory, therefore, the electrolytes, which are the substances capable of conducting the current when dissolved in water, are dissociated or split up by the water into two parts, or ions, which may be elements or more complex groups with the power of transmitting the current through the solution.

These ions are not free atoms or molecules, as they would in that case act as the element does; but they are particles heavily charged with electricity, which, upon loss of their charge, are set free as atoms. These ions have the power to transmit the current, the amount carried being constant for each ion, depending upon its constitution; that is, an ion of hydrogen has the power to transmit a certain amount of electricity, and this is the same for every and all hydrogen ions, while those of sodium, for instance, have a different constant carrying capacity. As might be expected, from the fact that hydrogen is the lightest element and diffuses more rapidly than any other, we find that its ions show the greatest rapidity of migration. The action taking place in a dilute solution can, perhaps, be best explained by means of a simple example. If we dissolve sodium chloride in water we have ions of sodium with a charge of positive electricity and ions of chlorine with a charge of negative electricity. As the solution becomes more concentrated the ions, through loss of their charges by the neutralizing action of those of an opposite nature, become atoms and unite with the atoms of the other element to form molecules, so that the product isolated by evaporation is identical with that introduced in the first instance. In order, therefore, to gain any knowledge of the nature of these ions we must study the substance in solution in water. If the sodium were in the atomic condition in the solution, it would, of course, decompose the water and liberate the hydrogen. As long as no current is passed through the solution no change is noticed, as the movements of the ions are equal in all directions and there is no tendency for an accumulation at one point. If, however, a current of electricity is passed through the solution the direction of movement is influenced by the current, and the sodium ions will accumulate at one pole and the chlorine ions at the other. The positive charges of the sodium ions will be neutralized by the negative charge on the adjoining pole, and the atomic sodium thus liberated will act on the water and liberate hydrogen. At the other pole, in a similar manner, the chlorine ions will lose their charge and escape as free chlorine. The fact is probably well known to physicians that pure water is not readily decomposed by the electric current; but if a little sulphuric acid is added the decomposition takes place even with a very weak current. How can we explain this phenomenon, where a small amount of sulphuric acid suffices to cause the decomposition of a large amount of water without itself suffering any loss? It can be readily explained by the electrolytic dissociation theory. The sulphuric acid in the water is dissociated into H ions and $SO_4$ ions. Now when the current passes the charge on the hydrogen ions is neutralized and the hydrogen set free. At the other pole, where there is an accumulation of $SO_4$ ions, the water is decomposed by the $SO_4$ group when it loses its charge and the hydrogen of the water combines with this group to form sulphuric acid, while the oxygen is liberated. Many phenomena which could not easily be explained before the discovery of these facts are now better understood, and we see why the inorganic salts which are ordinarily so stable, but which are easily dissociated in water solution, should be more active in solution than the unstable organic compounds which do not dissociate in water solution.

This theory has been of great assistance to the analytical chemist, as he can now explain many of the reactions which are so familiar, but whose cause was not known before. The general method of testing for the presence of soluble chlorides in water is to add a solution of silver nitrate to the suspected sample, and if it contains the chloride, a precipitation of silver chloride takes place. We have in one solution ions of potassium and ions of chlorine, or as they are generally written, Naions and Clions, and in the other Agions and $NO_3$ions. When these two solutions are brought together the Ag ions and the Cl ions combine to form insoluble AgCl, after they have lost their charges, and the Na ions and $NO_3$ ions remain in solution. If, however, instead of NaCl we use $NaClO_3$, sodium chlorate, we do not have a formation of insoluble AgCl as the second ion is not Cl, but a more complex group containing chlorine, $ClO_3$. We would conclude from this that a silver salt can only precipitate the chlorine when the latter is in the ionic condition. We thus see how the views as to the nature of solutions have entirely changed, and it may be of interest to the homœopathist to see how these views confirm the ideas of Hahnemann as to the greater activity of substances in the dilute form. The knowledge which we now have of the action of substances in solution will enable us to explain facts which he determined empirically, and we see how such scientific studies may have an important bearing on the theories of Homœopathy. As the activity of the inorganic and many organic compounds in solution is dependent upon the amount of dissociation which has taken place, and as this in turn increases with dilution, we see, perhaps, some reason for the efficacy of a high potency in many cases where a lower one will not act. Reasoning again from the studies of solution, we would expect the triturations made in water to be more effective than those made in alcohol, for we know that water is the strongest dissociating agent known. That the free ions in a dilute solution have important physiological action is shown by the work of Kahlenberg and True upon the toxic action of dissolved salts. Their experiments were made with plants, and similar results have been obtained with animal organisms, so we have reason to believe that the action towards the higher animals would be of the same general character. They showed that the plants are poisoned and killed by the free ions in certain cases, and they studied the connection between the number of ions present and the action on the plants. They showed that no matter what acid was used the amounts of free ions necessary to poison the plant were equivalent in every case, and they also showed this action to be due to the hydrogen ions. In one case mentioned 1 part of ionic hydrogen to 6,400,000 of water, corresponding to 0.00056 per cent. of hydrochloric acid, was sufficient to kill the plant. Metallic ions were found to be very poisonous, and a strong point in favor of the action being due to free ions was that while copper ions in the proportion of 1 part of copper to 400,000 of water killed the plant, another solution, in which the copper was present in a complex ion, and not as a single ion, could be made so strong that it had a blue color and contained about sixty times as much copper as the other solution, without killing the plant.

Silver was also found to be very poisonous, as one part of silver to 948,148 of water was sufficient to kill the plant. Facts of this kind cannot fail to impress one with the important *role* that the ions play, and while, no doubt, many effects will be ascribed to them, with which they have no connection, it will not affect the value of such observations as these. There is a tendency to enlarge and extend generalizations far beyond the point that can be sustained by experimental proof, and it is only necessary to recall such cases as the sensation which arose from the statements made by followers of Koch with regard to the value of his lymph as a remedy for consumption. The wild claims that are made in such cases come from those who are not familiar with the subject, and are startling as compared with the strongest statements of the discoverer, who has, perhaps, spent the greater part of his life in close touch with the subject. There is a tendency in the human mind to follow lines of speculation until one is lost in the maze and is astonished at the results of his deductions; but it has been the aim in this paper to avoid such fields, and to lay emphasis on those points which seem to be supported by a firm foundation of experimental proof.

